# Knowledge, Access, and Decision-Making: Women’s Financial Inclusion In Pakistan

**DOI:** 10.1145/3209811.3209819

**Published:** 2018-06-20

**Authors:** Samia Ibtasam, Lubna Razaq, Haider W. Anwar, Hamid Mehmood, Kushal Shah, Jennifer Webster, Neha Kumar, Richard Anderson

**Affiliations:** University of Washington Seattle, WA, USA samiai@cs.washington.edu; Information Technology University Lahore, Pakistanlubna.razaq@itu.edu.pk; Northeastern University Boston, MA, USA anwar.h@husky.neu.edu; Information Technology University Lahore, Pakistanhamid.mehmood@itu.edu.pk; University of Washington Seattle, WA, USA kushals@uw.edu; University of Washington Seattle, WA, USA jenniweb@cs.washington.edu; Georgia Institute of Technology Atlanta, GA, USA neha.kumar@gatech.edu; University of Washington Seattle, WA, USA anderson@cs.washington.edu

**Keywords:** ICTD, Financial Inclusion, Gender, Pakistan, Digital Financial Services, Women

## Abstract

In resource-constrained economies, lack of financial participation prohibits women’s economic empowerment and opportunities to improve circumstances. With the advent of Digital Financial Services (DFS), a growing emphasis has been placed on the possible positive impact of DFS on lives of individuals. However, for people to understand, adopt, and use DFS, they require certain prerequi-sites and enablers. In this paper, we use a mixed methods approach to analyze the gendered barriers in the readiness for and adoption of DFS as well as the impact of gendered roles in curtailing or enhancing the same. We present our analysis of 51 semi-structured interviews to evaluate the affordances or, lack thereof, in afford-ability of funds, authority of transactions, access to technological devices, and agency of social and cultural mobility–all of which are prerequisites to fully utilizing DFS. We discuss the sociocultural and religious context in Pakistan that underpins some of these gendered barriers and the perceived views of both men and women.

## 1 INTRODUCTION

In this paper, we draw attention to the impact of gendered roles in Pakistan that support, enhance, or limit the readiness for and adoption of Digital Financial Services (DFS) by Pakistani women. Before talking about the specific context of Pakistan, we situate the topic in the global perspective.

Around two billion people worldwide do not have a formal financial account, and more than 50% of the world’s poorest households are unbanked, which means that they lack formal financial services like savings, investments, and insurance [51]. This can be the result of insufficient penetration of the banking network or the absence of collateral or proper documentation of users, leaving those users at risk to slip back into poverty with a single financial shock as well as exposing them to exploitative financial mechanisms.

The concept of financial inclusion refers to creating solutions which enable access to financial services for all members of a society, particularly those at the bottom of the economic pyramid [51]. DFS aim to address the problem of financial inclusion stemming from insufficient banking infrastructure in emerging economies [4]. They provide mechanisms for accessing savings, spending, and utilization of money digitally [29] through mobile money platforms, retail agents (Over-The-Counter or OTC transactions), and mobile phone-based services.

Resource-constrained economies, like Pakistan, face additional challenges to achieving financial inclusion. Globally, there is a gender gap, however, this gap is amplified in emerging economies where women and children suffer disproportionately. This gap also manifests in lacks of financial capacity, empowerment, and security. In developing economies, 37% of women are banked compared with 45% of men. This gap is further increased for rural women who have a 28% lower chance of owning a bank account than men [31]. We define gender as the culturally and socially constructed differences between men and women that vary from place to place and time to time [40]. These differences also include perceptions and self-efficacies about women’s capabilities to use technology [52] and their financial decision making [44].

In this paper, we report a mixed-method study of 50+ semi-structured interviews with individuals from different geographic and diverse socioeconomic backgrounds in Punjab, Pakistan as well as statistical data from the Financial Inclusion Insights (FII) Survey 2016 for Pakistan. We provide a nuanced view of the factors that influence whether and how these individuals have the prerequisites to use DFS and the role of gender in access, agency, and mobility for financial transactions. Our research confirms, and in some cases, controverts the typically assumed barriers that limits women’s understanding and use of DFS.

### 1.1 Motivation

Although Pakistan is considered a DFS ready country [19, 49] with its strong national identification system, steadily increasing smart-phone and internet penetration, and branchless banking friendly regulations [6, 50], it has a low financial inclusion rate, which is even lower for women. The lower financial inclusion of women may be the result of Pakistan’s gender gap, which, in 2016, the World Bank reported as second to last (144 out of 145 countries) in their Gender Gap report [55].

Given the persistent concerns about gender equity in Pakistan, we find that the path to women’s inclusion is afflicted by a host of challenges that persist across all aspects of everyday life [23]. Lack of uptake of financial services can, therefore, be a cumulative result of the gender disparities in the prerequisites to adopt and use these services. These disparities may manifest in the realms of owning and using technology, earning and spending income, understanding and practicing complex financial behaviors, and physical mobility. The goal of our research is to understand the role of gender and the resulting affordances, barriers, and opportunities it provides especially in financial knowledge, transaction, and decision making of women. The long-term goal is to identify how the needs and gendered dynamics affect financial matters, and where and how digital financial services can (and cannot) support the women of Pakistan.

## 2 RELATED WORK

### 2.1 Technology and Gender

Studies on differences in technology use, knowledge, or access based on gender not only form the basis of our work but are also ex-tended by our work. [9] discussed how phone use and access varies by gender, demonstrating how men and women show different pat-terns of phone use even when spending the same amount of time on the phone, and [34] outlined gendered patterns of communication over mobile phones, banking, and internet use. Specifically, in the context of Pakistan, [45] investigated gender disparities between men and women through an analysis of cell phone logs. Researchers have made attempts to evaluate the reasons behind use and non-use of mobile phones by women, such as the perceived lack of technical knowledge [54].

Research on mobile access and usage in low and middle-income countries, including Pakistan, indicates the existence of a gender divide [16, 57]. According to [17], the gender divide is primarily influenced and framed by socio-economic and political factors, which also include social and cultural barriers to the use of technology. [56] studied Kenyan women’s interactions with mobile devices and argued that HCI and ICTD researchers should focus on designs specific to the everyday needs of women rather than trying to overcome socioeconomic concerns. While technology has been shown to be a source of gender empowerment for women in the case of mobile phone usage [18], other studies have found certain mobile phone functions, such as text messaging, to be limiting for women who do not possess certain skills of literacy and numeracy [13]. Through ethnographic work in Brazil, [38] explored how men and women adopt new technologies and found that gendered spaces are associated with technology adoption. [41] investigated multiple marginalities and their impact on technology accessibility. They proposed that gender is a major factor in how technology is accessed and that women utilize technology to overcome gendered institutional and social barriers. In terms of design interventions, [3] implemented Protibadi, a mobile phone app, as a way for women to have a voice about public harassment.

### 2.2 Digital Financial Services & Financial HCI

While financial services have appeared within ICTD research in the past, the initial studies focused on microfinance [15, 43] and microcredit services [42] on feature phones [33]. Studies have since addressed the various barriers that limit the use of financial services by women [5], including factors like lack of documentation, collat-eral, and agency to use financial services [30]. Research also shows that the rural and gender gaps in financial inclusion are persistent throughout all developing economies [12].

DFS have been analyzed both from a technical perspective [10, 11] and from a social perspective [46]. Researchers have also explored how technology can support the usage of DFS through designing user interfaces that enable money transfer [32] and improve the learnability of money transfer smartphone applications [20]. [37] made the case for a focus on financial education because, in addition to technology, policy and regulation can affect adoption and use.

The field of financial HCI is nascent as indicated by [25]. Our work extends the research in this area as financial HCI looks at both human interactions around money as well as the associated technological artifacts and the impacts of one on the other. Al-though finance remains a very secretive and private concept [26] there has been research on how people perceive, manage, and utilize money. For example, [53] looked at how low-income people manage money in England and how technology helped or hindered in managing finances. [39] studied loan workflows with both digital and real money and how social meanings, social relations, and socio-technical ecosystems impact the practice of dealing with money. [27] did an ethnographic study of the existing payment and banking practices to identify the applications and services to support the needs of the community.

### 2.3 Gender and Culturally Situated Design

Gender as an analytical category for HCI has appeared in recent literature [7]. Beyond this, many researchers have elected to engage in a feminist HCI framework to guide their research and ultimately to inform how they think about technology design. This framework has focused HCI researchers on the importance of and sensitivity to gender identities [8]. It has also given them an appreciation for the usefulness of value-sensitive design [14]. Furthermore, it has enabled researchers to apply and engage with feminist HCI in areas like public safety [24], computing, and design [1].

We also emphasize that technology adoption and use are more likely to succeed if researchers consider what is meaningful in the daily lives of their target users. In terms of culturally situated design, HCI researchers are beginning to consider how religious institutions influence people’s technology use and adoption [47]. While religious institutions may offer one avenue for context-specific de-sign, understanding how interpretations of religion affect the daily lives of people, including perceptions of gendered roles, may also lead to fruitful design recommendations.

## 3 METHODOLOGY

Before embarking on our field work, we identified the various elements that collectively impact the adoption of DFS. These include 1) affordability, which means that one has funds (from any source) for transactions and 2) authority, which suggests permission and agency to use these funds and / or ownership of the funds necessary to make transactions. Without affordability and authority, individuals cannot make financial transactions. An additional element includes 3) access to sources of DFS transactions including technology to use DFS (phones and SIM for wallets) or access to agent shops (OTC transactions). Without access to or availability of technological devices, as well as the know-how to use them, funds can only be transferred using non-digital means. Thus, we consider the interplay of affordability, authority, and technology access as the primary lens for our inquiry.

Through this lens, we inquired about the understanding and performance of gender roles as well as the socio-cultural and economic conditions that affect affordability, authority, and technology access. In our analysis, we explored the limitations and barriers faced by women due to perceived gender roles, the various sociocultural and gendered reasons for these barriers, the uses and non-uses of financial technologies due to mobility and ownership, and design opportunities that can be utilized to enable DFS usage and adoption among women in Pakistan.

### 

#### 3.0.1 Financial Inclusion Insights

Our research takes a mixed methods approach, drawing from an analysis of both qualitative interviews and an existing quantitative survey, Financial Inclusion Insights (FII) [22]. FII tracker survey is a periodic survey of national scale conducted annually in eight countries to measure financial inclusion status as well as the knowledge and information level of the population [21]. We reviewed the publicly available dataset of the third wave of FII for Pakistan, which contained a total of 6000 data points for various metrics. We observed trends for financial inclusion among both genders, which better allowed us to understand the Pakistani context of financial inclusion.

### 3.1 Qualitative Fieldwork

Prior to conducting our fieldwork in the province of Punjab, Pakistan, we had several assumptions about the impact of socioeconomic conditions for women and what these meant in terms of women’s empowerment in general and, more specifically, women’s financial inclusion. The assumptions that aided us in our recruitment criteria are:

A1 -Financial empowerment and financial income are not correlated. We based this assumption on local contextual information that indicated that some women, who do not earn, still have access to funds in the forms of finances provided by their families or close relatives. In contrast, other women with income sources have little to no authority to decide how that money is allocated.

A2 -Education is not directly correlated with the financial empowerment of women. Many of the limitations that are based on socioculturally determined gendered roles remain unaltered by the education level of the women or that of their family members.

Considering these assumptions, we elected to interview women from all varieties of socioeconomic and cultural backgrounds. We did not assume that any particular woman was more financially em-powered than another. We also interviewed several men to discuss their perspective on gendered roles and their impact on financial empowerment.

#### 3.1.1 The Interviews

The interviews of women were conducted by women who were fluent in English, Urdu, and Punjabi. Like-wise, male researchers interviewed male participants. Following the culturally and socially accepted gendered norms facilitated a level of comfort amongst participants who then felt at ease to disclose personal information and accounts. We conducted interviews in the preferred language of the participants, recorded the interviews with participants’ consent, and later made transcriptions. The interviews ranged from 45-60 minutes depending on the participants’ responses and were conducted in the participants’ preferred location (i.e., participants’ homes, participants’ offices, office buildings, homes where they worked, microfinance offices, and vocational training centers). We transcribed and encoded the interviews to evaluate the common themes as well as additional findings that went beyond or contradicted the barriers described in previous literature.

The semi-structured interviews consisted of two sets of questions. The first set was close-ended questions related to literacy, income level, access to finances, ownership of and access to phones, SIMs, bank accounts, and the household in general. This first set was to gauge if participants were below the basic threshold of affordability (of money) and access (to phones), and, thus, could not benefit from technological interventions at this time. The second set was open-ended qualitative questions that allowed the participants to further detail their experiences.

#### 3.1.2 The Participants

In summer of 2017, we conducted a total of 51 semi-structured interviews of both men and women in and around the cities of Lahore, Multan, and Kasur and their connecting villages. We spoke with 41 women and 10 men, in urban, periurban and rural parts of northern Punjab (34 interviews) and southern Punjab (17 interviews). The women (ages 15 and older) varied from non-literate to well-educated (university graduates). Table 1 illustrates the demographic summary of the participants.

**Table 1:** Participant Demographics

Gender	Men	Women
Participants	10	41
Age	18 -25 yrs: 5	18 -25 yrs: 16
	26 -45 yrs: 25	26 -45 yrs: 3
	>46 yrs: 2	>46 yrs: 0
Locality	Urban: 4	Urban: 22
	Peri-Urban: 3	Peri-Urban: 7
	Rural: 3	Rural: 12
Marital-Status	Unmarried: 5	Unmarried: 17
	Married: 5	Married: 24
Education Level	Uneducated: 3	Uneducated: 11
	Up to 8th grade: 3	Up to 8th grade: 5
	Up to 12th grade: 0	Up to 12th grade: 9
	College: 4	College: 16
Profession	Employed: 9	Employed: 26
	HouseHusbands: 0	HouseWives: 6
	Entrepreneur: 1	Entrepreneur: 9

We relied on purposeful sampling using our social contacts and also worked with the microfinance organization, Akhuwat, that lends small-scale loans to households and entrepreneurs. A large number of their borrowers are women, who run beauty parlors, embroidery shops, and other small businesses. When visiting the organization’s centers in Kasur (North) and Multan (South) we were introduced to some of their clients. We recruited participants through word of mouth and relied on Akhuwat to reach participants in areas where we could not openly recruit. None of the participants were given incentives or were reimbursed for their participation.

Some of the women worked outside their homes as maids, high-income professionals, office staff, assistants, teachers, beauticians, nurses, software developers, and entrepreneurs. Others were house-wives, some of whom had previously worked or ran their own entrepreneurial enterprises from within their homes (i.e., jewelry makers, home-based beauty parlors, and embroiderers). All participants had access to funds either in the form of their salary, income from their businesses, and / or money given to them by their husbands or family members (or they at least knew about the finances being managed by the male members), and, thus, could answer questions about financial decision making.

### 4 FINDINGS

In this section, we present our findings on women’s social, technological, and financial access in Pakistan using our analysis of qualitative interviews and FII data.

#### 4.1 Social Access And Gender

The gender gap in Pakistan manifests not only as differences in access to physical assets and material opportunities for men and for women but also as the ability to choose how to manage and benefit from those assets. Offering a mobile phone or a financial services infrastructure alone cannot address the problems of women’s financial inclusion without the ability to make decisions and take actions on one’s own behalf.

**Mobility**

Financial services, like frequent trips to a bank to access services, require frequent mobility. However, DFS has the benefit of shifting most interactions to a mobile device with less frequent need to leave one’s home. To open a mobile wallet account or top up one’s wallet or cash out, one has to visit an agent shop, which could be a small grocery store in the nearby market. Impediments to mobility because of cultural, religious, or security concerns can, therefore, hamper a woman’s ability to access financial services even if those services are digital.

We observed that women possess various degrees of mobility, which we categorize as being forbidden to go outside of the home, as permitted to leave with a male relative, or as permitted to travel alone. From our qualitative interviews, we learned that the preferred days or times of travel are driven by the mobility category to which women belonged. Respondents who had to be accompanied preferred times that were most convenient for their male relatives, which included either after work or weekends. Women who traveled alone preferred to do so early in the day or mid-day when they were free from domestic chores or when it was relatively cooler outside.

Pakistani society endorses gender segregation in both public and private settings. Interactions with unknown men can be both atypical and stressful for women, especially in the case of unsolicited contact. Women informed us that when they traveled alone, they faced problems such as longer waits before finding a ride, men following them to their homes or blocking their way, verbal and physical harassment, and incidents of theft and mugging on public transport. This is true for women in other developing countries as well [3, 24]. Therefore, women traveling alone preferred private transport or rickshaw. Women disliked public transport fearing physical and verbal harassment, traveling with strangers, and longer travel times. For low-income women, the lack of affordability of private transport made physical mobility even more difficult. One woman said that, “ I don’t prefer Chingchi because anyone can hop on and off, and it gets really uncomfortable if a guy gets on.”,(female, urban). Another woman specified that “There are many issues that we have to face while going outside the home. People tease girls in public transport and markets but it’s better to stay quiet, finish your work, and return home without paying attention to anyone.” (female, urban)

Previous negative experiences of women made them more vigilant and selective about the places they would visit. Most women shared that they made conscious choices of visiting markets or public places with more people to avoid harassment. Even then, places with mostly male visitors were avoided (e.g., mobile shops for buying airtime or phone repair). Women, who had previously felt comfortable traveling alone, altered their behavior after being faced with incidents of harassment or crime. One woman related the following: “I was once mugged while walking. So, I don’t go around window shopping anymore.”, (female, urban)

Concerns for physical safety are the dominant constraint in women’s ability to move freely, and these concerns are exacerbated at night, further driving the necessity for accompaniment by a male relative. None of the participants, irrespective of the permission status for traveling alone, education, or working status, preferred or felt secure going outside after dark on their own. One woman expressed her fears in this way: “There are many issues in going out at night. Sometimes when a kid gets sick and we need to take him to the hospital at night, it becomes very difficult. There is a risk of robbery or mobile or bike snatching while traveling at night.” (female, urban)

Traveling permissions originated from male relatives and were influenced by concerns for women’s safety as well as the social perception of the family. From our interviews, we observed that although traveling permissions changed for some women after marriage this did not hold true for all married women. Participants resoundingly indicated that being accompanied by a male relative made travel safer. For instance, this woman said that “I go with my husband if I need to go out in the evening or at night. It’s not safe to go out in the evening and it’s also considered bad in our culture [for women to travel alone].” (female, rural). Another woman explained that “No, I never go outside of the house without my husband’s permission. And if it’s an emergency then I tell him and go with him or if he says go with my son, I go with my son” (female, rural). Both women and men, when describing their concerns about women traveling alone -be it their sisters, wives or daughters-focused more on socio-cultural apprehensions rather than on personal preference. Even though interpretations of Islam in South Asia encourage purdah (seclusion from unrelated men), participants did not mention this as a reason for the prohibition of movement. For instance, one woman said that “I don’t let my daughter go anywhere alone. There are many relatives and neighbors out in the street. They might say that her daughter is roaming around alone. I pick and drop her at the stitching center.” (female, rural)

#### 4.2 Religious Interpretations

**Religion and Gender**. Being an Islamic majority country, the social and cultural norms in Pakistan are influenced heavily by interpretations of Islam. Islamic law indicates functional roles based on gender. Men are responsible for acting as the Head of the House-hold (HOH), earning income, and providing for the family. This is intended to ensure financial security for women. Women are entitled to support as daughters, wives, mothers, and sisters. Similarly, women hold the right to refuse to perform household chores (e.g., women can ask for money in return for nursing their babies). However, at the same time, men make decisions for the family and may inherit twice as much as women in order to fulfill these financial obligations (Quran 4:11). Thus, participants also derived their understanding and beliefs around gender from the interpretations of Islam that are prevalent today in Pakistan. They discussed how financial decision making, as denoted by Islamic law, assigns men the role of head of the household. One woman related that, “Fathers are the head of households in all of the families. We saw this before our father died. After his death, our mother started making decisions. In all other families, females are asked for suggestions. It’s good because men make good decisions and it’s their role. It is said that the house where women lead isn’t successful.” (female,periurban)

Some male participants referenced religion as the source from which they derived ideas about the family, including their responsibility to protect the family from the deterioration of society. In

our conversations we saw women who were completely comfortable with their husbands or fathers running the household with or without their consent or contribution because, as per the participants, the men were performing their duties to the household. One woman’s opinion was as follows: “Men should take the final decision. Because God has made it that way.” (female, urban) Another declared, “Men of the house are household heads. In our family, in other houses, my uncles (from the maternal and paternal side) make decisions. Islam says that men are accurate and gents know more things.” (female, urban)

**Religion and Finance**. Islamic legal discourse does not allow or encourage charging interest on loans. Prior to our fieldwork, we hypothesized that participants would state the involvement of interest in all bank dealings as a reason for non-use of formal financial services. However, in our conversations, only a few participants mentioned interest as a concern. We also noted that concerns about interest rate varied by affluence. Women with alternatives, who could forgo interest-based options by borrowing from family members etc, mentioned that interest-based options were not preferable. However, small-scale female entrepreneurs, who did not have enough resources, relied on interest. One woman’s concern with interest-based loans was purely economical. She lamented how she had experienced a business loss because of high-interest rates on microloans. This influenced her decision to later take out an interest-free loan from a microfinance institution.

### 4.3 Technological Access

When women have limited mobility, due to seclusion or other issues discussed earlier, mobile-based propositions may provide them with access to previously unavailable information and services. This is especially advantageous in limited banking infrastructure settings. The current DFS paradigm requires phone ownership with a SIM registered in an individual’s own name. This forms the basis of the Digital Transaction Account for that particular individual and provides benefits such as the notion of identity as being linked to a verification process or a certain device for executing transactions.

**Phone Access**. In Pakistan, mobility constraints for women are accompanied by a significant gender gap in mobile ownership. The FII survey data shows that women’s ownership of mobile phones (36.7%) is approximately half that of men (79.4%). Phone owner-ship is also distinctly lower in rural areas compared to urban areas. Although 35 of 41 women participants had phone access, as mentioned in Table 2, we explored the underlying reasons why women had not own phones both in the past and in the present. Most women considered ’permission’ as a limiting factor to ownership or access. Our interviews also revealed that phone ownership among unmarried women was considered inappropriate due to concerns about maintaining an image of purity to ensure future marriage prospects [23]. The element of permission, or its lack thereof, is even more influential in single women’s phone ownership. One woman explained that, [w]omen are not allowed to use phones in our family. They just do not feel the need when men have them [phones]. I have a phone, but most people know that it is my mother’s. My father, however, is less restrictive. My brothers are more restrictive. My brothers say that if they have a mobile phone, I can borrow it from them. I should not have my own mobile.(female, periurban)

**Table 2:** Technology access, ownership and usage

Gender	Men (10)	Women (41)
Phone Access	Yes: 10	Yes: 35
	No: 0	No: 6
Access Type	Personal: 10	Personal: 31
	Shared: 0	Shared: 4
Phone Type	Feature: 3	Feature: 9
	Smart: 7	Smart: 26
Sim Registration	Self: 10	Self: 22
	Others: 0	Others: 9
Internet Usage	Yes: 8	Yes: 26
	No: 2	No: 15
Internet Access	Wi-Fi (W): 0	Wi-Fi (W): 4
	Cellular (C): 4	Cellular (C): 8
	W & C: 4	W & C: 14

We also determined that the constraints of non-use by unmarried women were based on hearsay of women eloping with men who had contacted them by phone. Our interview responses confirmed that male relatives dictated phone ownership as well as the type of phone (feature phone or smartphone). These constraints depended on the men’s technical education and familiarity with technology. While some men prevented the use of phones altogether, others insisted that feature phones could be used but not smartphones, and some restricted functions like social networking applications or uploading of photographs. One participant expressed that, “I gave my brother money to buy me a smartphone. But he brought me a feature phone, asking, what would I do with a smartphone. (female, urban)”

**Phone Ownership**. Only a few women shared that they them-selves bought the phones. Mostly, phones were purchased by male relatives, with or without consultation regarding women’s phone preference. In rural regions of southern Punjab, a few women re-ported receiving phones (either a phone or an upgrade to a smart-phone) as gifts from their husbands at their wedding. One newlywed recounted that, “[m]y husband gifted me a phone on our wedding. I did not have a phone before this. In our family, unmarried girls are not allowed to have a phone. I, my mother-in-law, my sister-in-law, all of us use this phone. When my husband comes home, my sister-in-law (unmarried) puts down the phone. Otherwise, she plays games on it continuously!” (female, peri-urban). The marriage did not ensure phone use for every woman. For example, one woman described her situation as follows: “No it wasn’t about permission. I had unmarried girls at home. So my sons did not let me keep a phone. They used to tell me to get them married and then you can have a phone at home. (Why didn’t they let you?) No special reason. The environment of the household is not like this. Now they [the daughters] are thankfully married. So I also got it [a phone]. And my sons too!” (female, rural)

One participant reported that she did not begin using the phone she received from her (female) employer before seeking permission from her husband. In general, we noted that the freedom to engage digitally was curbed for several reasons. However, these limitations were not imposed on women alone; they were also imposed by women. For example, several women participants indicated that they used the internet on their tablets, laptops, or their husbands’phones. Yet, they were careful not to buy internet access for their own phones to prevent their children from using it as illustrated here: “I use it on my husband’s phone if he has the internet package. I use the internet for exploring things for my beauty parlor and stitching. I don’t use internet on my phone because I don’t want kids to use the internet. If I would have the internet package, kids will start using it because they unlock my phone and keep using it.” (female,peri-urban) Most male participants believed that there are dangers or disadvantages to women using mobile phones with some citing social or personal preferences. One male participant (peri-urban) confided that his father had recently passed away and that he was now responsible for taking care of this entire family. He did not permit his younger sister to own a phone, even though his elder sister had owned a phone before marriage when his father was alive. Another participant (urban) said, “Even on YouTube and other such apps, un-solicited vulgar images or videos appear, which are inappropriate for women to see.” On further inquiry, he indicated that it was okay for men to view these videos, but not women. A few male participants said that they do not try to restrict their female relatives and that they feel reluctant to share their annoyance about women’s use of technology as well. These men had wives from a socioeconomic class in which they could not restrict their wives’ technology use. However, their opinions aligned with those that favored restrictions. One participant (rural) said that women’s ownership of phones only increases their likelihood of talking to male strangers, and, thus, it should not be allowed (starting from a very early age). Another participant (peri-urban) shared that he had allowed his daughter to use a tablet because she was using it in front of him at home, yet he did not agree to buy a SIM for the tablet.

**Digital Harassment, Fraud and Theft**. Concerns about harassment, which discourage women’s mobility in public spaces, are also present in the digital space. When faced with harassment, women relied on the male relatives to address the issue. One participant described over the phone harassment: “I used to get harassment calls. A delivery man got my number and then he would call. I didn’t file any complaint but I told it to my husband. He handled it.” (female, urban)

Beyond harassment, men were concerned about women being more susceptible to socially engineered frauds and how phones increased women’s chances of falling prey to fraudsters. However, rather than educating or informing women about potential frauds, men suggested banning the use of phones altogether. One man exclaimed that, “I don’t want them [women] to talk to anyone who can use them or who can gain money through fraudulent scams, either through having an affair or otherwise. Someone can ask them for help over the phone. Women have not seen the world and they just don’t know who to trust.” (male, urban)

The lowest income rural women reiterated what their male elders had emphasized the dangers of phones for women. One woman from a rural area outlined her concerns: “Yes, women can have more issues [than men]. It is easy to snatch a mobile from them. However, if someone places a gun to your head, anyone would give up their mobile. But women have jewelry and other stuff.” (female, rural). In contrast, the lowest income urban participants thought that phones could be useful for women and were resentful of the lack of access or ownership. Many women also claimed that phones had both pros and cons, yet they believed that the pros outweighed the cons, and, thus, the use of phones should not be banned for women. Some women were aware of how a phone might open up possibilities: “Mobiles have numbers of so many organizations that you can reach out to, for many issues. Females are strong these days.” (female, peri-urban)

Women from rural communities who worked were not allowed cell-phones. Thus, their business-related calls and orders were received on their husbands’ phones. A rural midwife explained that, “For seven months now, I’ve delivered babies with a lady doctor. The calls for deliveries come through my husband’s phone. I am not allowed to own a phone even though I am allowed to go (for deliveries).” (female, rural)

#### 4.4 National ID Cards and SIMs

The government of Pakistan mandates every adult (above 18 years) to have a Computerized National ID Card (CNIC). Pakistan has a strong national identification system (NADRA), with reportedly 90% of its population registered in a central identification system [49]. Although 83% of our female respondents owned CNICs, some respondents informed us of reasons why they or the women in their families previously did not have CNICs. The reasons included missing documents required for CNIC (e.g., birth certificates or marriage certificates), long travel distances, long queues, and the inability to pay for the new required SIM-based CNICs.

In Pakistani villages it is common practice to ask a village guard who has basic literacy to record the Date-of-Births (DOBs) of newborns. People refer to this DOB when requesting a CNIC. However, such bookkeeping is prone to errors as the guards often forget or mix-up names and dates, or are relocated. One woman, whose DOB was recorded by a guard, told us that due to imprecise bookkeeping of her DOB and that of her sister, she could never get an ID since the system would not accept DOBs recorded with only three months between the births of the two siblings. She could not exist in the National database, which made it impossible for her to open a bank account and prompted her to use her sister-in-law’s name for a SIM as well as other family members’ names on other official documents.

Frequent security checks, in the last two decades, require Pakistani citizens to produce their CNIC, which has a direct effect on mobility. This has motivated the acceptance of CNICs among citi-zens.Women informed us that they had to produce their ID cards in certain residential areas, and because of this, they are necessary. Other reasons for obtaining a CNIC included marriage and the transfer of property. A mother explained her reasons for obtaining CNIC: “I got a CNIC because my son needed me to have one before he could get his. They demand the CNIC of the father and mother for issuing a child’s CNIC. My son has to pass through an army check post while going to work and they don’t let him pass without a CNIC.” (Female, Rural)

Obtaining a SIM card in Pakistan requires having a valid CNIC, where one person can have a maximum of five SIMs. In 2016, due to heightened security requirements, the Interior Ministry of Pakistan mandated biometric verification of all SIM cards owners [35, 36]. The FII dataset shows that (97%) of men and (92.2%) of women SIM owners successfully verified their SIMs.

During interviews, we asked women about their SIM ownership and biometric verification. Contrary to the unpopularity of SIM verification in Bangladesh [2], many women stated that after the enactment of the new legislation, male relatives urged the women to transfer SIMs to their national IDs, thus avoiding penalties for having more than five SIMs. According to the FII data, some women have SIMs registered in someone else’s name. Having such SIMs operational means they have been verified with the name of the household member rather than the woman actually using them. In our interviews, some women had SIMs in the name of their husbands, brothers, mothers, or sisters-in-law. A few participants shared how their family did not think transferring a SIM to their CNIC was necessary. One woman described her situation as follows: “No this SIM is in my mother’s name. I don’t have a need for my own number. I use my mother’s number. My brother doesn’t give me permission either. My brother asks, ’why do you need a SIM?’ There are so many issues in Pakistan because of ID cards.” (female, urban). In contrast, all the male participants had SIMs registered in their own name.

**Financial Services And Inclusivity**

#### 4.5 Financial Authority and Head of Household

In Pakistan, men are typically considered the heads of households (HOH) and are assumed to be the primary earners. This emphasizes the importance of male offspring and also determines the roles of both men and women in terms of finances and authority. In our interviews, none of the 41 women claimed to be the HOH, as mentioned in Table 3. The HOH was usually the father or another male elder, a husband, or a brother. Whereas, three of the ten male respondents claimed HOH status.

We also saw that the lower the social status and income level of the family, the higher the need for financial participation from women. One woman explained her situation as follows: “My father-in-law is the head of the house. He manages all these things like bringing something from the market like vegetables and other things but sometimes he gives money to me for shopping and asks me to go and bring the required things. My husband and his brothers give their salary to their father. But the amount that we earn from embroidery or beauty parlor work is ours and we don’t need to give it to our father-in-law.” (female, peri-urban). Another shared: “My husband keeps our money with him, in his pocket. It’s not safe to keep the money at home as our house is on rent and then I and my husband stay away from the home all day and kids are alone at home.” (female, urban) Women do not necessarily have control over their earned funds and, in turn, save without the knowledge of their male relatives. Women from both urban and rural locations practiced saving money privately as illustrated here: “I keep some of my savings in a locker at the vocational training institute where I study embroidery. I don’t tell my brother about this money. Otherwise, he will ask me to give all of the money to him.” (female, urban) and here: “I save money. But one doesn’t have to tell men. Today my daughters are young, but tomorrow they will grow up so quickly.” (female, rural)

**Table 3:** Participant’s household and financial setting

Gender	Men (10)	Women (41)
Supplementary		Husband: 16
Financial Source	None: 10	Parents: 5
		None: 20
		Job: 7
		Husband: 16
		^Job & Husband: 4^
Household or Personal Spending Source	_Job: 5_	^Parents: 7^
	_Business: 4_	^Sister: 1^
	_None: 1_	Male relative: 2
		Rent: 1
		None: 3
Participant as		
	Yes: 4	Yes: 0
household head	No: 6	No: 41
		Parents: 15
		Husband: 14
		In-laws: 3
Other household heads	_Parents: 6_	^Brother: 1^
		Mother &
		Brother: 1
		Husband & Self
		both: 7

#### 4.6 Making Transactions and Dealing with Money

In our interviews, we asked about the details of both large and small financial decisions to understand the dynamics of financial decision-making. We also found a correlation between gender and decision making based on the amount in question. For example, one woman stated that “My dad thinks that electronics are big financial decisions, so they (girls) should take advice.” (female, urban).

Since women are not allowed to move freely outside of their homes, they rely on secondary methods of transacting even with the available finances. Low-income women participants were only allowed to go to and from work, but they were required to be accompanied by other women who worked nearby. These women only purchased items that were available on their way to work. Women participants relied on their male relatives and children to buy groceries for them. One such woman outlined this process as follows: “I send my third-oldest child with a daily grocery list. Sometimes we have money and sometimes we don’t have money to pay the shopkeeper. So we are on khaata [diary, meaning credit] with the shopkeeper”, (female, rural)

#### 4.7 Bank account ownership

According to FII, 12% of men and 6.4% of women in Pakistan own a bank account. We also observed this pattern in our interviews with 50% of men owning an account compared to 34% of women. The FII also showed higher bank account ownership in urban areas (13.4%) over rural (7.4%), which is expected due to limited banking options in rural areas. Apart from the overall disparity between urban and rural banking penetration, the gender disparity also persisted across this urban-rural divide with 19% of urban men and 8.6% of women owning bank accounts as compared to 9.3% of rural men and 5% of rural women. None of the 12 rural women we interviewed had bank accounts.

The FII data shows that in Pakistan more men (98%) make transactions themselves than women (77%) and that 23% of women rely on someone to assist them. In our interviews, most, if not all, women said that they did not go to banks. Women in urban areas sent their husbands, office boys, or trusted colleagues for bank transactions. In peri-urban and rural settings, women preferred to not have a bank account because there was no need, money, or permission. One woman outlined her experience with banks as follows: “When my husband used to work in Lahore, he used to leave the checkbook with me. Sometimes when someone was coming home, he would give them money; sometimes he would come himself. Sometimes I used to give a check to the kids to go to the bank and cash. I have never been there [to the bank] by myself. Only one time, my son took me to pay the bill, because he said that the queue for women is shorter at banks.” (female, rural) Another woman said she had frustrations with going out and that it was easier to leave the account in her husband’s name. She said that, “I do not have an account on my name because I don’t go out of the home and it’s better to save in my husband’s account because he can go anytime to deposit or withdraw. If I go I will have to answer many questions from my father-in-law like, ’Where are you going? Why are you going?’ (female, peri-urban)”

Although some participants preferred keeping money at home due to easy access, others kept it in a savings groups or at a bank. Outside of the usual savings in banks, some participants saved secretly in banks to hide money from family. Men and women would sometimes collaborate to overcome the mandates of the HOH, as in this case: “He [participant’s husband] has a bank account. We haven’t told anyone at home about this account. My father-in-law asks us to give all of our money to him to manage. But we have kids and we should have our own savings. We keep a balance in the bank account that we get from committee [savings group] but we never tell anyone about this.” (female, peri-urban)

#### 4.8 Mobile Money

In the FII data, a total of 9% of respondents used mobile money including both accounts and OTC transactions, with 14% of males and only 4.5% of women using it. The FII survey inquired about mobile money services from respondents without distinguishing the level of knowledge or usage. Thus, we asked participants about mobile money services available in Pakistan (e.g., brand recognition), and their knowledge of mobile money, the source of knowledge, and previous experience. Eight of the ten men had used mobile money accounts at least once, and the majority of women had not used a mobile money account. The FII data shows that men engage in more activities on their mobile money accounts and do so more frequently than women. Many women recognized the EasyPaisa and Jazz Cash brands, the two largest mobile money players in Pakistan, but most participants did not know the details of transaction costs or the whereabouts of nearby shops that provided OTC services. Most of the women said that they knew about these services from their male relatives or had overheard of their existence in conversations.

We asked women about the possibility of mobile money shop-keepers (OTC agents) defrauding them. Urban and peri-urban women who had heard of mobile money seemed to trust the OTC agents. One urbanite considered: “I don’t think a shopkeeper can defraud you. It is the responsibility of the shopkeeper [to send money]. He began this service at his shop. If he commits fraud, he will have to close his shop.” (female, urban). However, rural women feared the complexity of the transaction and questioned giving money to a shopkeeper: “I do not trust any of these services. What if we send money to someone but the shopkeeper doesn’t make the transaction? Or when we go to the shop and there is someone else there. Who will we contact with a dispute in such a situation…I have heard that shopkeepers abuse and slap women.” (female, rural). Contrary to this, men from rural areas, described ways to assure a successful transaction: “To assure (that the money has been transferred) I make a phone call, right there, standing in the shop. It’s only human to be skeptical.” (male, rural)

The State Bank of Pakistan recently mandated biometric verification of the recipient of a mobile money transaction before disbursement of funds (previously a secret code was required). In the past, women had sent male relatives to OTC shops to receive money in-person. One female participant knew about this change, explaining that “[i]n the modern era, we have biometric verification. Instead of sharing a secret code, you only have to place your thumb and they give you money.” (female, rural). This can either increase a woman’s mobility or also reduce transactions initiated or sent to a woman’s account. However, this will potentially help with avoiding frauds like the one described here: “Previously shopkeepers used to tell their friends the secret pins and their friends used to receive money. Now there is biometric verification.” (male, rural)

#### 4.9 ROSCAs or Savings Groups

Savings Groups or Rotating Savings and Credit Associations (ROSCAs), commonly known as ’committees’ in Pakistan, are a social saving activity which is also popular in many other parts of the world [48]. All women participants knew about ROSCAs and had participated at some point in their lives (with the exception of a few high-earning urban women). Most women had grown up hearing about or seeing other people, especially mothers and other female relatives, participate in ROSCAs. One such woman shared that, “I have saved money through committees since I was a teenager. I like saving money through committees. My mother used to manage committees, but that required large amounts of money, so I used to have a share in one committee with three or four other members.” (female, urban) Men had heard of ROSCAs, but only one of the men we interviewed had directly participated and another one participated indirectly via his mother. One interviewee shared that he did not feel comfortable participating in ROSCAs due to a large number of women.

Most women relied on the administrator or the person running the group for assurance and trust. Whereas some women said that they participated in the groups only if they knew everyone, or if the other members were relatives, family friends, or neighbors. Women told us about the existence of daily, weekly, and monthly ROSCAs, ranging from PKR 10 (USD 10 cents) to a few thousand (USD 100-500). One teenage girl had bought her family a used fridge and her brother a used computer by saving in a PKR 10 (10 cents) daily ROSCA.

Most women began participating in larger or more regular ROSCAs after marriage when they realized the need for savings and planning ahead. We observed habitual participation in ROSCAs, goal-oriented saving through ROSCAs, and special occasion ROSCA participation due to an upcoming expense (e.g., weddings). One ROSCA participant explained that, “I used to save money in committees in the past. My husband and I both participated and we each contributed one thousand per month. From the money that we got from the committee, we purchased some land with installment payments. Now the installments are complete.” (female, rural). Women who currently borrowed with the microfinance loan organization shared that they no longer participate in ROSCAs because they do not have discretionary income to do so.

### 5 Additional Findings And Recommendations

Here, we include additional findings that will influence the direction and design of services for women, in particular, Digital Financial Services (DFS).

**Influential women**. During our field work, we came across women who had influence over other women as employers, vocational trainers, entrepreneurs, and women with higher levels of education or technology exposure. These women could be recruited as technological and social ambassadors for other women. While many of these women are already consulted passively, we argue that they could also actively inform other women. The literature mentions that women require trustworthy sources of information as well as frequent assurance. These women could encourage their employees and relatives to learn about, own, and experiment with technology. Women’s comfort with trusted groups for financial saving might also be leveraged to provide additional financial information and advice.

**Go where the women are**. We observed that although women, both users, and non-users, were aware of the location of the nearest banks, they had hardly noticed the existence of mobile money shops. Bank branches place great stock on visibility compared to a multi-purpose corner shop. We propose increasing the visibility of mobile money shops as a step to enable more participation by both men and women. We also recommend to bring the mobile money services to places which are frequented by women such as beauty saloons or neighborhood shops for them to be more accessible for women.

**Women entrepreneurs as DFS-ready**. Women entrepreneurs who run small businesses (e.g., beauty parlors, embroidery shops, boutiques, and textile and clothing vendors) overcame certain cultural norms regarding mobility and agency. They also enjoyed greater access to and ownership of phones in addition to managing funds. Beyond this, some of our women entrepreneur participants sought out information on the internet on their device or a male relative’s device. The women in this group are already well-positioned and often encouraged to seek more financial and technical solutions to improve their work, which indicates that their businesses could align well with future initiatives to design DFS services for women.

**Marital status affects access**. We observed that social trends regarding the ownership of mobile phones or mobility among women are linked to their marital status and are affected by assumptions regarding the effects of owning mobile phones on marriage prospects for young girls. However, the situation often changed when a woman married, and she was allowed a phone, given an upgraded phone, or permitted to additional mobility.

**Women need more financial services**. ROSCAs, MFIs, and OTCs have served women’s financial needs in various ways from savings to small loans to remittance, however, they have limitations. ROSCAs and OTCs lack records to evaluate individual credit history. With ROSCAs, women expressed concerns that they did not receive any more money in return than they had contributed. Microfinance loan approval takes a long time and often comes with the disadvantage of high-interest rates. OTCs are not the same as mobile wallets wherein an account is created, and, thus, a record of financial activity. With such a record, credit is more readily assessed, opening up possibilities for new options to save, request loans, and explore other services such as insurance. Financial inclusion of women remains a problem because women may need to be approached differently, but are currently only exposed to the version of products and services designed for the population at large. Service providers consistently fail to see women as a niche market with a unique set of circumstances, behaviors, and requirements.

**It is believed that men know better**. Men are often perceived to have more capacity to understand issues, and are consulted by women to solve problems (financial, technological, or social). In Pakistan, this problem-solving structure can be attributed to interpretations of Islam that underlie many of the constraints faced by women. In our observation, we did, however, see some variation of the roles of women in their financial decision-making and freedom of movement.

**Women can teach future generations**. Many of the practices reported by our participants, both financial and otherwise, were learned from their family members, especially mothers. All of the women we interviewed had learned about ROSCAs from their mothers. This suggests that other financial mechanisms or tools, when explained to women by other women, especially mothers, could have a significant impact on their uptake and use.

### 6 Discussion

In the preceding sections, we share the various technological, social and financial access of Pakistani women in urban, peri-urban and rural settings. Our findings indicate that gender plays an important role in deciding financial capability and decision-making, techno-logical access, and physical movement of individuals. This, in turn, affects women’s abilities to make decisions and take actions that lead to the adoption and use of DFS.

Affordability, authority, and access are all necessary and a lack or absence of any of these three, causes limitations in the access to, understanding, or use of DFS among women. Some women we spoke to had insufficient funds due to limitations of technology access and/or gendered roles that gave men financial authority in their families. In other instances our findings demonstrate socio-cultural influences that result in women giving up authority, funds or mobility (access).

The men of the household make decisions about women’s physical movement and technology ownership. People consider unmarried women who venture out of the home and use phones as putting their family’s reputation at risk due to the fears that they will speak with unrelated men. Male relatives monitor women closely and are widely viewed as a source of authority.

Our findings illustrate the many intricate personal, social, and cultural reasons that underlie the gender-based restrictions and limitations for women in Pakistan. We try to understand the affordances and limitations, self-imposed or otherwise, and in doing so, explore the implications of gender on financial authority, transactions, and decision making. The findings paint a nuanced picture of women’s demographics, education, social standing, professional level, earnings, and marital status as well as the men in their lives (from fathers, brothers, uncles, husbands to even sons) playing a role in the technological, social, and financial flexibility available to women. In addressing problems faced by women, it is not only important to understand and address concerns of women, but also to take into consideration the concerns of those who make decisions for the women, including men [28]. Most importantly, women cannot be clustered as 50% of the population and rather are diverse individuals with varying socio-cultural and economic setups. These diverse conditions cannot be simplified in one solution and need to be studied individually.

Mobility poses a key impediment to access to services by women, including financial services like frequent visits to banks or OTC shops. Women reported that they had to seek accompaniment by men of the household to visit shops or banks and that the women who traveled alone often faced harassment and were constrained by the scheduling of domestic duties.

In revisiting the two assumptions about women’s financial income and education that we put forward in the methodology section, based on our fieldwork, we would argue that both of these assumptions hold. Many socio-cultural and gendered barriers exist that transcend income level, social class, and education level of women. We categorize the constraints mentioned by our participants as short-term and long-term barriers, where short-term barriers include technological solutions (e.g., support in the privacy of transactions from the men in their lives and women-specific value propositions). Some of the constraints mentioned by women are ingrained in sociocultural and religious interpretations. We do not propose to question or change these, but point out that some of the factors limiting women’s participation in financial services and adoption of DFS are long-term barriers that cannot be solved by technology alone. These gendered barriers pose open questions for future directions of research in DFS and for ICTD in general.

### 7 Conclusion

We conducted this study using a combination of gender-segregated quantitative analysis of the Financial Inclusion Insights (FII) Wave 3 dataset on Pakistan and 51 qualitative interviews of 10 men and 41 women to assess the constraints in women’s adoption of DFS and affordances to create such access. In this paper, we outline the various sociocultural, religious, and gendered dynamics that influence Pakistani women’s abilities to access technology as well as to afford and authorize financial transactions. We also share the many nuances of the numerous constraints that impact women. Finally, we identify certain women who may be more readily receptive or able to adopt context-specific DFS design for women.
